# Mitochondrial Variation in *Anopheles gambiae* and *Anopheles coluzzii*: Phylogeographic Legacy and Mitonuclear Associations With Metabolic Resistance to Pathogens and Insecticides

**DOI:** 10.1093/gbe/evae172

**Published:** 2024-09-03

**Authors:** Jorge E Amaya Romero, Clothilde Chenal, Yacine Ben Chehida, Alistair Miles, Chris S Clarkson, Vincent Pedergnana, Bregje Wertheim, Michael C Fontaine

**Affiliations:** Groningen Institute for Evolutionary Life Sciences (GELIFES), University of Groningen, Groningen 9747 AG, Netherlands; MIVEGEC, University of Montpellier, CNRS, IRD, Montpellier, France; MIVEGEC, University of Montpellier, CNRS, IRD, Montpellier, France; Institut des Science de l’Évolution de Montpellier, University of Montpellier, CNRS, Montpellier, France; Groningen Institute for Evolutionary Life Sciences (GELIFES), University of Groningen, Groningen 9747 AG, Netherlands; Ecology and Evolutionary Biology, School of Biosciences, University of Sheffield, Sheffield S10 2TN, UK; Wellcome Sanger Institute, Hinxton, Cambridge CB10 1SA, UK; Wellcome Sanger Institute, Hinxton, Cambridge CB10 1SA, UK; MIVEGEC, University of Montpellier, CNRS, IRD, Montpellier, France; Groningen Institute for Evolutionary Life Sciences (GELIFES), University of Groningen, Groningen 9747 AG, Netherlands; Groningen Institute for Evolutionary Life Sciences (GELIFES), University of Groningen, Groningen 9747 AG, Netherlands; MIVEGEC, University of Montpellier, CNRS, IRD, Montpellier, France

**Keywords:** *Anopheles gambiae* complex, mitogenome, mitonuclear coevolution, phylogeography, insecticide resistance, OXSPHOS

## Abstract

Mitochondrial DNA has been a popular marker in phylogeography, phylogeny, and molecular ecology, but its complex evolution is increasingly recognized. Here, we investigated mitochondrial DNA variation in *Anopheles gambiae* and *Anopheles coluzzii*, in relation to other species in the *Anopheles gambiae* complex, by assembling the mitogenomes of 1,219 mosquitoes across Africa. The mitochondrial DNA phylogeny of the *Anopheles gambiae* complex was consistent with previously reported highly reticulated evolutionary history, revealing important discordances with the species tree. The three most widespread species (*An. gambiae, An. coluzzii*, and *Anopheles arabiensis*), known for extensive historical introgression, could not be discriminated based on mitogenomes. Furthermore, a monophyletic clustering of the three saltwater-tolerant species (*Anopheles merus*, *Anopheles melas*, and *Anopheles bwambae*) in the *Anopheles gambiae* complex also suggested that introgression and possibly selection shaped mitochondrial DNA evolution. Mitochondrial DNA variation in *An. gambiae* and *An. coluzzii* across Africa revealed significant partitioning among populations and species. A peculiar mitochondrial DNA lineage found predominantly in *An. coluzzii* and in the hybrid taxon of the African “*far-west*” exhibited divergence comparable to the interspecies divergence in the *Anopheles gambiae* complex, with a geographic distribution matching closely *An. coluzzii*'s geographic range. This phylogeographic relict of the *An. coluzzii* and *An. gambiae* split was associated with population and species structure, but not with the rare *Wolbachia* occurrence. The lineage was significantly associated with single nucleotide polymorphisms in the nuclear genome, particularly in genes associated with pathogen and insecticide resistance. These findings underline potential mitonuclear coevolution history and the role played by mitochondria in shaping metabolic responses to pathogens and insecticides in *Anopheles*.

## Graphical Abstract

**Graphical abstract:**
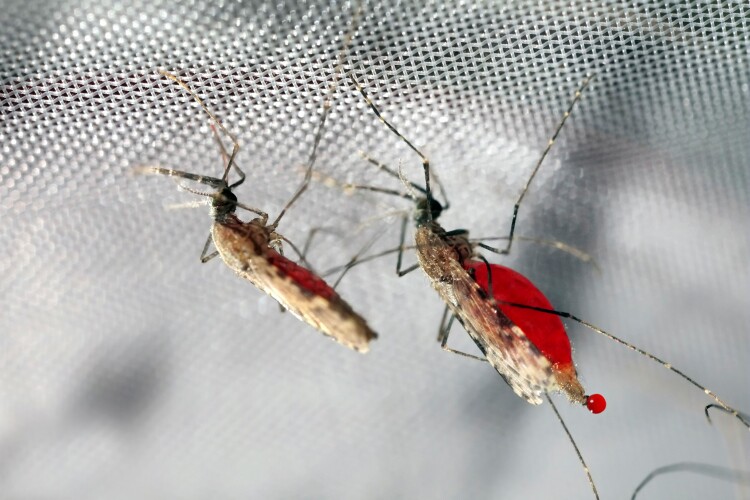
*Anopheles gambiae*. Credit IRD - Patrick Landmann, Vectopôle, Montpellier.

**Significance**

This study explores the determinants of mitochondrial (mtDNA) genetic variation in *Anopheles gambiae* and *Anopheles coluzzii*, key African malaria vectors, and other related species of the *Anopheles gambiae* complex (AGC). By analyzing the mitogenomes of 1,219 mosquitoes across Africa, we uncovered a remarkable diversity with significant phylogeographic partitioning among species and populations, widespread mtDNA introgression among AGC species, possibly with adaptive value, and supporting the previously reported highly reticulated evolutionary history. A particularly divergent mtDNA lineage predominantly observed in *An. coluzzii* (and the hybrid taxon) suggests a phylogeographic relict of species isolation and secondary contact. This lineage's association with nuclear genes linked to pathogen and insecticide resistance highlights potential intricate mitonuclear coevolution. These findings underline the complex determinants of mtDNA evolution in *Anopheles* mosquitoes, and the role mitochondria may play in adaptive responses to pathogens and insecticides, providing crucial information for developing more effective malaria vector control strategies.

## Introduction

Historically, mitochondrial DNA (mtDNA) has been among the most popular genetic markers in molecular ecology, evolution, and systematics. Among its applications are assessing population and species genetic diversity, genetic structure, phylogeographic and phylogenetic patterns, species identity, and metabarcoding (Galtier et al. 2009; Dong et al. 2021; Dowling and Wolff 2023). Contributing factors to such popularity include an easy access to the mtDNA genetic variation compared with nuclear markers, even in degraded tissue samples, due to the large number of per-cell copies. Likewise, its haploid nature and clonal inheritance through the female germ line provide an account of evolution independent, and complementary, to the nuclear DNA's (nuDNA). Because the mtDNA does not recombine, the entire molecule behaves as a single segregating locus, with a single genealogical tree representative of the maternal genealogy (although rare exceptions have been reported; Zouros et al. 1994; Saville et al. 1998; Vissing 2019). Furthermore, its reduced effective population size together with an elevated mutation rate compared with the nuclear genome makes the mtDNA a fast-evolving, and potentially highly informative genetic marker (Galtier et al. 2009; Allio et al. 2017; Dong et al. 2021; Dowling and Wolff 2023). At the same time, however, the fact that mtDNA is a single nonrecombining locus limits its power to describe the evolutionary history of populations and species.

Another argument in favor of mtDNA's popularity as a genetic marker is its near-neutrality and constant mutation rate, but an increasing number of studies now contend that selection and other factors can significantly impact mtDNA variation and its evolution (Bazin et al. 2006; Galtier et al. 2009; Dong et al. 2021; Dowling and Wolff 2023). Indeed, mtDNA evolution in arthropods and especially insects can be significantly impacted by cytoplasmic incompatibilities (CIs) with endosymbionts like the *Wolbachia* bacteria (Hurst and Jiggins 2005; Galtier et al. 2009; Dong et al. 2021; Dowling and Wolff 2023). Furthermore, epistatic interactions between mitochondrial and nuclear genome are also suspected to modulate mtDNA genetic variation given the key biological processes happening in the mitochondria and the tight coordination between the two genome compartments (Wolff et al. 2014; Sloan et al. 2015; Rand et al. 2018; Dowling and Wolff 2023; Nguyen et al. 2023). For example, an increasing number of studies in mosquitoes suggests that mitochondrial respiration and the associated production of reactive oxygen species (ROS) play a significant role in mosquito immune response and metabolic processes involved in pathogens and insecticides resistance (Van Leeuwen et al. 2008; Ding et al. 2020). These epistatic interactions are often neglected in ecology and evolution due to the limited number of studies with adequate datasets to test for these effects. The determinants of mtDNA variations in mosquitoes can thus be multifarious (Hurst and Jiggins 2005; Bazin et al. 2006; Galtier et al. 2009; Cameron 2014; Wolff et al. 2014). Therefore, in many cases, mtDNA does not follow a simple neutral genetic evolution and its use in molecular ecology, metabarcoding, and phylogeographic studies requires a clear assessment of the various factors potentially influencing its evolution. However, doing so necessitate investigating mtDNA variation in combination with nuclear genomic data. This is now increasingly possible thanks to democratization of whole-genome resequencing and large-scale genomic consortium projects.

The genomic resources provided by two consortia—the MalariaGEN *Anopheles gambiae 1000 genome (Ag1000G)* consortium (The Anopheles gambiae 1000 Genomes Consortium 2017, 2020) and the *Anopheles* 16 genomes project (Neafsey et al. 2013, 2015; Fontaine et al. 2015)—offer a unique opportunity to explore the determinants of mtDNA variation in two sister mosquito species within the *Anopheles gambiae* species complex (AGC): *Anopheles gambiae* and *Anopheles coluzzii*. The AGC is a medically important group of at least nine closely related and morphologically indistinguishable mosquito sibling species (White et al. 2011; Coetzee et al. 2013; Barrón et al. 2019; Loughlin 2020; Tennessen et al. 2021). Three members of this African mosquito species complex (*An. gambiae*, *An. coluzzii*, and *Anopheles arabiensis*) are among the most significant malaria vectors in the world, responsible for the majority of the 619,000 malaria-related deaths in 2021, 96% of which occurred in sub-Saharan Africa and impacted primarily children under the age of five (World Health Organization 2022). The ecological plasticity of these three species contributes greatly to their status as major human malaria vectors (Coluzzi et al. 2002). In contrast to the other AGC species (*Anopheles quadriannulatus, Anopheles merus, Anopheles melas, Anopheles bwambae, Anopheles amharicus*, and *Anopheles fontenllei*) with more confined geographic distributions, these three species have wide overlapping distributions across diverse biomes of tropical Africa. This ecological plasticity in the AGC is attributed to a large adaptive potential, stemming mainly from three major genomic properties: (1) a strikingly high number of paracentric chromosomal inversion polymorphisms segregating in their genome, which are implicated in adaptation to seasonal and spatial environmental heterogeneities related both to climatic variables and anthropogenic alterations of the landscape, in phenotypic variation such as adaptation to desiccation, or even resistance to pathogens like *Plasmodium* sp. or insecticides (e.g. Coluzzi et al. 2002; Costantini et al. 2009; Simard et al. 2009; Cheng et al. 2012; Ayala et al. 2017; Riehle et al. 2017; Cheng et al. 2018); (2) an exceptional level of genetic diversity identified in natural populations provides a rich material onto which natural selection can act (The Anopheles gambiae 1000 Genomes Consortium 2017, 2020); and (3) a high propensity for hybridization and interspecific gene flow connecting directly or indirectly the gene pools from all the species over the evolutionary timescale of the complex, and especially between the three main malaria vectors (*An. gambiae*, *An. coluzzii*, and *An. arabiensis*) (Crawford et al. 2015; Fontaine et al. 2015; Thawornwattana et al. 2018; Müller et al. 2021).

The species of the AGC radiated within the past 400 to 500 kyr (Thawornwattana et al. 2018; Müller et al. 2021), and the speciation barriers are not yet fully formed. Although all members of the AGC can be crossed in the laboratory and produce fertile female hybrids but sterile male hybrids (except for *An. gambiae* and *An. coluzzii*), interspecific hybridization rate in nature is supposed to be extremely low (<0.02%) (Pombi et al. 2017). An exception is the two sister species *An. gambiae* and *An. coluzzii* which diverged more recently (between 40 and 60 kyr before present) according to the most recent estimates (Thawornwattana et al. 2018; Müller et al. 2021). These two sister species are at an earlier stage of speciation with no postzygotic isolation detected (reviewed in Pombi et al. (2017)). Hybrid offsprings of both sexes are viable and fertile in the laboratory, but strong prezygotic and premating isolation barriers have been identified in nature. The hybridization rate is low (*ca.* 1%) across their overlapping distribution range in West Africa, even if high hybridization rates (up to 40%) were reported in the populations from the African “*far-west*” (i.e. the coastal fringe of Guinea-Bissau and Senegambia (estuary of the river Gambia and Casamance in Senegal) (Lee et al. 2013; Nwakanma et al. 2013; Pombi et al. 2017; Vicente et al. 2017). New evidence suggests however that these “hybrid” or “intermediate” populations from the African “*far-west*” could be a putatively distinct cryptic hybrid taxon in which diagnostic alleles typically used to discriminate between *An. gambiae* and *An. coluzzii* are still segregating (Caputo et al. 2024). Nevertheless, despite the low occurrence of contemporary hybridization rates between the members of the AGC, the large geographic overlap in species distributions, together with porous reproductive barriers have resulted in extensive levels for interspecies hybridization over the evolutionary timescales (Fontaine et al. 2015).

Extensive introgression rates between species of the AGC combined with elevated levels of incomplete lineage sorting (ILS) due to large effective population sizes contributed to maintain high levels of shared polymorphisms and highly discordant phylogenies along the nuclear genome, greatly hampering the identification of the species evolutionary history (Crawford et al. 2015; Fontaine et al. 2015; Thawornwattana et al. 2018; Müller et al. 2021). Genome-scale studies depicted a highly reticulated evolutionary history of the AGC with outstanding levels of geneflow being detected between the two sister species—*An. gambiae* and *An. coluzzii*, and also between *An. arabiensis* and the ancestor of *An. gambiae* and *An. coluzzii*. Most of the genomic regions resistant to introgression on their nuclear genome, and thus informative on the species branching order, were mostly identified in the X chromosome, and scattered across less than ∼2% of the autosomes (Crawford et al. 2015; Fontaine et al. 2015; Thawornwattana et al. 2018). The lack of any obvious phylogenetic patterns of species structure at the mitochondrial genome further supported the extensive level of introgression between these three species (Caccone et al. 1996; Besansky et al. 1997; Fontaine et al. 2015; Hanemaaijer et al. 2018). Additional interspecific introgression signals were also detected between *An. merus* and *An. quadriannulatus*, between *An. gambiae* and *An. bwambae*, and also along the ancestral branches of the AGC species (Thelwell et al. 2000; Crawford et al. 2015; Fontaine et al. 2015; Thawornwattana et al. 2018; Müller et al. 2021). Although the selective and evolutionary effects associated with this extensive level of introgression between species of the AGC remains to be fully investigated, clear evidence of adaptive introgression were detected involving chromosomal inversions (Fontaine et al. 2015; Riehle et al. 2017; Thawornwattana et al. 2018) and insecticide resistance loci (Clarkson et al. 2014; Grau-Bové et al. 2020, 2021; Lucas et al. 2023).

**Fig. 1.**
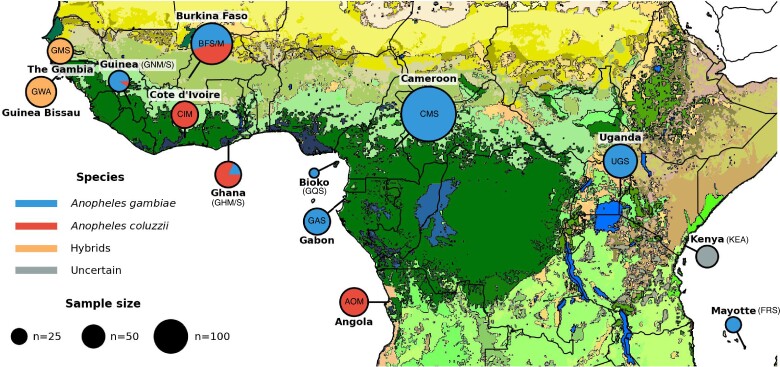
Approximate sampling locations and sample size per location of the 1,142 samples of *An. gambiae* and *An. coluzzii* from the The Anopheles gambiae 1000 Genomes Consortium (2020). The population codes are also provided within or next to the pie charts (see Table 1 and supplementary table S2, Supplementary Material online). Colors within the pie charts describe the species and include: *An*. *gambiae* (formerly the *S-form* of *An. gambiae*), *An. coluzzii* (formerly known as the M-form of *An. gambiae*), the hybrid taxonomically uncertain populations of the African *far-west*, and the taxonomically uncertain population of Kenya. The figure is modified from The Anopheles gambiae 1000 Genomes Consortium (2020). Map colors represent ecosystem classes; dark green designates forest ecosystems. For a complete color legend, see Fig. 9 in the work of Sayre (2013) (see supplementary table S2, Supplementary Material online for further details on the sampling).

Here, we leveraged the genomic resources from The Anopheles gambiae 1000 Genomes Consortium (2017, 2020) phase-II and from the *Anopheles* 16 genomes project (Neafsey et al. 2013, 2015; Fontaine et al. 2015) to explore the determinants of mitochondrial genetic variation in the AGC with a particular focus on *An. gambiae* and *An. coluzzii*. For that purpose, we first assembled mitogenomes for 1,219 pan-African mosquitoes (Fig. 1 and supplementary fig. S1, Supplementary Material online) using a new flexible bioinformatic pipeline, called *AutoMitoG* (*automatic mitogenome assembly*) (supplementary fig. S2, Supplementary Material online), which relies on *MitoBIM* approach that combines mapping and de novo assembly of short-read sequencing data (Hahn et al. 2013). We then assessed the level of mtDNA variation, its phylogeographic and population structure, and the mtDNA genealogical history in relation to the population demographic history previously estimated from the nuclear genome (The Anopheles gambiae 1000 Genomes Consortium 2017, 2020). We further assessed what factors may best explain the mtDNA phylogeographic structure, testing various covariates including *Wolbachia* infection status, population structure estimated from nuclear genome data, and chromosomal inversions. Finally, we investigated the mitonuclear associations that possibly suggest coevolution and coadaptation between the two genomic compartments.

## Results and Discussion

### The *AutoMitoG* Pipeline and Assembly of the Ag1000G Mitogenomes

The *AutoMitoG* pipeline, which streamlines mitogenome assembly using the *MitoBIM* approach (see Materials and Methods section and supplementary fig. S2, Supplementary Material online), successfully assembled 1,219 mitogenome sequences from the unmapped short-read data originating from the two *An. gambiae* consortia projects (Fontaine et al. 2015; The Anopheles gambiae 1000 Genomes Consortium 2017, 2020; Fig. 1 and supplementary fig. S1 and tables S1 and S2, Supplementary Material online). We first assessed the pipeline performance by comparing newly assembled mitogenome sequences with those from the 74 samples of the AGC previously generated in Fontaine et al. (2015) (supplementary fig. S1 and table S1, Supplementary Material online). Average assembly length before any trimming and sequence alignment was 15,366 base pairs (bp). Following Fontaine et al. (2015) and after sequence alignment, we removed the control region (CR) resulting in a 14,843 bp alignment length. Excluding the CR removed most of the ambiguities and gaps remaining in the alignment (supplementary fig. S3, Supplementary Material online). The previous and present bioinformatic pipelines generated very similar mtDNA assemblies for each sample with one exception (samples ID: Aara_SRS408148, supplementary fig. S4, Supplementary Material online). Beside that sample which resulted from a label mistake in the DRYAD repository of Fontaine et al. (2015), mitogenome sequence pairs for each sample were nearly identical with a number of nucleotide differences of 0.4 on average (25% quartile: 0.0; 75% quartile: 1.0, max: 3.0) (supplementary fig. S4, Supplementary Material online). We augmented this alignment with newly assembled mitogenome sequences from three *An. bwambae* samples of lower sequencing quality than the other samples (supplementary table S1, Supplementary Material online). These mitogenomes assembled with the two pipelines also generated similar sequences with a slightly lower sequence identity (>99.9%) for each pair of assemblies, except one sample (bwambae_4) which was more difficult to assemble (supplementary figs. S4a and S5 and table S1, Supplementary Material online). Overall, the *AutoMitoG* pipeline performed at a good bench mark level.

We then applied the *AutoMitoG* pipeline to the 1,142 *An. gambiae* and *An. coluzzii* mosquito samples of The Anopheles gambiae 1000 Genomes Consortium (2017) (Fig. 1 and supplementary table S2, Supplementary Material online). Assembly lengths were 15,364 ± 1.9 bp on average (min: 15,358—max: 15,374) (supplementary table S2, Supplementary Material online). The raw alignment was 15,866 bp long and 14,844 bp after removing the CR and gaps. The 1,142 mtDNA sequence alignment of *An. gambiae* and *An. coluzzii* included 3,017 polymorphic sites (*S*), of which 1,195 singleton sites (*Sing.*), and a nucleotide diversity (*π*) of 0.004, defining 910 distinct haplotypes (*H*), with a haplotype diversity (HD) of 0.999 (Table 1).

**Table 1:** Mitochondrial genetic diversity statistics per species and population for the entire mitogenome alignment (14,844 bp)

Species	Group	Location	*N*	*S*	*Sing.*	Shared *P.*	*K*	*H*	*HD*	*π*	Tajima's D*	*N* (%) Cryptic
All	All	All	1,142	3,017	1,195	1,822	57.5	910	0.999	0.0039	−2.50*	232 (20%)
Hybrid	Hybrid	Hybrid	156	942	468	474	53.8	131	0.997	0.0036	−2.23*	77 (49%)
*An. coluzzii*	*An. coluzzii*	*An. coluzzii*	283	1,298	541	757	60	237	0.998	0.0040	−2.24*	89 (31%)
*An. gambiae*	*An. gambiae*	*An. gambiae*	655	2,336	1,004	1,332	54.3	539	0.999	0.0037	−2.51*	66 (10%)
*–*	Cryptic L.	Cryptic	232	983	505	478	32.2	199	0.997	0.0022	−2.55*	232 (100%)
*–*	Other L.	Common	909	2,719	1,051	1,668	53.9	713	0.998	0.0036	−2.53*	909 (0%)
*An. coluzzii*	AOM	Angola	78	323	121	202	37.5	50	0.983	0.0025	−1.48	4 (5%)
*An. coluzzii*	BFM	Burkina Faso	75	819	489	330	59.7	75	1.000	0.0040	−2.25*	37 (49%)
*An. coluzzii*	CIM	Côte d’Ivoire	71	552	260	292	58.6	58	0.988	0.0040	−1.71*	27 (38%)
*An. coluzzii*	GHM	Ghana	55	498	224	274	61.6	53	0.998	0.0041	−1.57*	20 (36%)
*An. coluzzii*	GNM	Guinea	4	61	61	0	30.5	2	0.500	0.0021	−0.87*	1 (25%)
*An. gambiae*	BFS	Burkina Faso	92	1,019	585	434	56.9	91	1.000	0.0038	−2.46*	10 (11%)
*An. gambiae*	CMS	Cameroon	297	1,537	639	898	56.6	217	0.996	0.0038	−2.41*	47 (16%)
*An. gambiae*	FRS	Mayotte	24	31	18	13	5.3	15	0.920	0.0004	−1.35	0 (0%)
*An. gambiae*	GAS	Gabon	69	283	117	166	38.3	48	0.972	0.0026	−1.23	2 (3%)
*An. gambiae*	GHS	Ghana	12	215	149	66	48.4	11	0.985	0.0033	−1.5	1 (8%)
*An. gambiae*	GNS	Guinea	40	574	357	217	57.3	38	0.997	0.0039	−2.16*	4 (10%)
*An. gambiae*	GQS	Bioko Island	9	78	48	30	22.8	9	1.000	0.0015	−1.06	0 (0%)
*An. gambiae*	UGS	Uganda	112	949	519	430	49.8	112	1.000	0.0034	−2.44*	2 (2%)
Hybrid	GMS	The Gambia	65	364	140	224	47.8	43	0.982	0.0032	−1.33	42 (65%)
Hybrid	GWA	Guinea Bissau	91	792	436	356	55.7	88	0.999	0.0038	−2.20*	35 (38%)
Uncertain	KEA	Kenya	48	81	1	80	32.3	4	0.650	0.0022	2.74	0 (0%)

Number of sequences (*N*), segregating sites (*S*), singletons (*Sing.*), shared polymorphism (*Shared P.*), average number of differences between pairs of sequences (*K*), number of haplotypes (*H*), haplotype diversity (HD), nucleotide diversity (*π*), Tajima's *D*, number (and proportion) of sequences belonging to the cryptic lineage (*N* (%) Cryptic).

^*^Stars on Tajima's *D* values indicate a significant departure from neutrality (*D*≠0) based on 1,000 coalescent simulations.

### Phylogenetic Relationships Among mtDNA Haplotypes Trace the Phylogeographic History of the Species Split and Introgression Among Species of the AGC

**Fig. 2.**
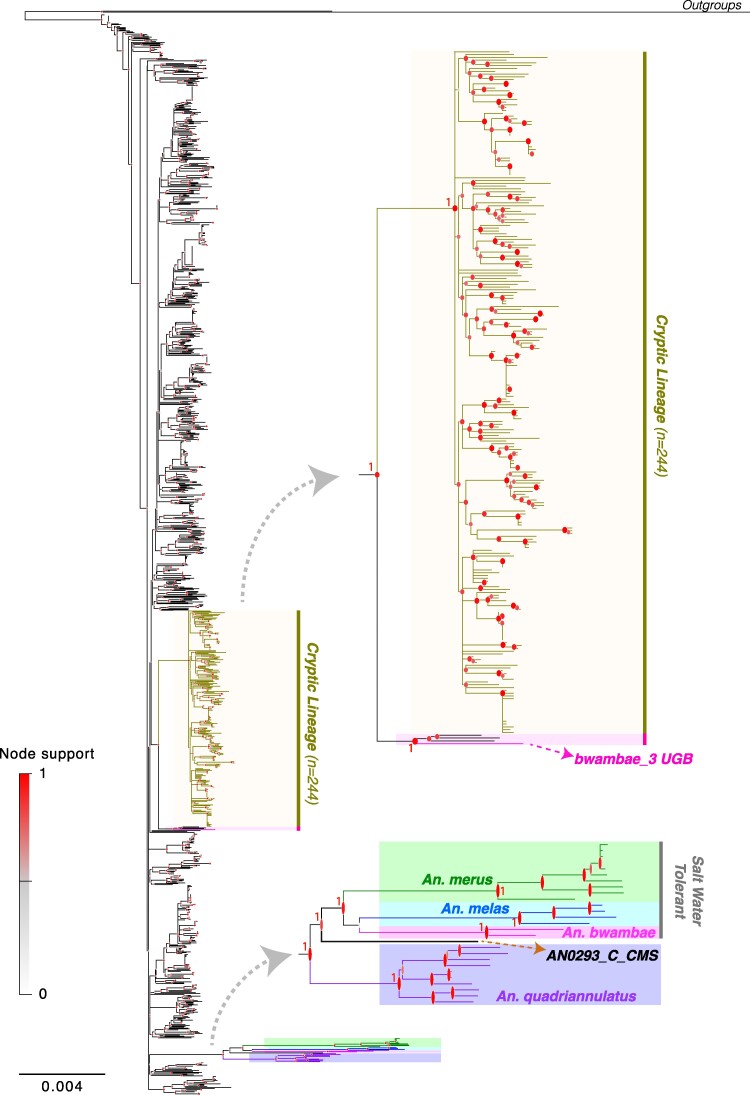
Phylogenetic relationships among mitogenome sequences. Maximum likelihood phylogeny estimated using PhyML based on the 1,222 mtDNA sequences composed of the 1,142 *An. gambiae* and *An. coluzzii* samples from *The Ag1000G Consortium*, 77 from the 7 species of the AGC from Fontaine et al (2015), one reference sequence from *An. gambiae*, and the two outgroups. (Black lines: *An. gambiae*, *An. coluzzii*, or *An. arabiensis*, Green: *An. merus*, Blue: *An. melas*, Purple: *An. quadriannulatus*, Pink: *An. bwambae*). High node support is indicated at the node with a red mark. Notice the absence of *An. arabiensis* specific mtDNA haplotype branching close to *An. quadriannulatus*. The cryptic lineage, highlighted in gold color, stands out of the genetic diversity of *An. gambiae*, *An. coluzzii*, or *An. arabiensis.* Also standing out are the four other species of the AGC. The subtree on the top right shows a zoom onto the cryptic lineage and its sister lineage among which stands the introgressed *An. bwambae* (bwambae_3) sample (in pink). The second subtree below focuses on the other species of the AGC displaying the monophyletic clustering of the saltwater-tolerent species (Green: *An. merus*, Blue: *An. melas*, Pink: *An. bwambae*). Highlighted is the presence of one *An. gambiae* individual (AN0293 CMS, orange arrow) next to *An. quadriannulatus*, in a place where *An. arabiensis* would be expected based on the admitted species tree (Fontaine et al. 2015).

Phylogenetic relationships among the 1,142 mtDNA sequences from *An. gambiae* and *An. coluzzii*, together with the 77 mtDNA sequences from the five other species from the AGC (Fig. 2 and supplementary figs. S5 and S7, Supplementary Material online) were consistent with previous studies. Indeed, previously reported evidence of extensive gene flow between *An. gambiae*, *An. coluzzii*, and *An. arabiensis* found support in our phylogenetic analyses with a complete absence of any mtDNA haplotype private to *An. arabiensis* samples (Fig. 2 and supplementary figs. S5 and S7, Supplementary Material online). From the mtDNA standpoint, the 12 samples of *An. arabiensis* could not be discriminated from *An. gambiae* and *An. coluzzii* as previously reported (Besansky et al. 1997; Donnelly et al. 2001; Fontaine et al. 2015; Hanemaaijer et al. 2018). Aside from *An. gambiae*, *An. coluzzii*, and *An. arabiensis*, the four other species of the AGC clustered in a divergent monophyletic clade (Fig. 2 and supplementary figs. S5 and S7, Supplementary Material online). Within that clade, the three saltwater-tolerant species (*An. melas*, *An. merus*, and *An. bwambae*) formed a monophyletic group next to *An. quadriannulatus* (Fig. 2 and supplementary figs. S5 and S7, Supplementary Material online). One *An. bwambae* sample (bwambae_3, Fig. 2, and supplementary figs. S5 and S7, Supplementary Material online) carried a mtDNA haplotype clustering among those from *An. gambiae*, *An. coluzzii*, and *An. arabiensis.* This is consistent with previous evidence of mitochondrial introgression between *An. bwambae* and one of these three species, most likely *An. gambiae* (Thelwell et al. 2000). Noteworthy, one *An. gambiae* specimen from Cameroon (AN0293_C_CMS, Fig. 2, and supplementary fig. S7, Supplementary Material online) carried a unique mitogenome haplotype closely related to, yet still divergent from *An. quadriannulatus*. The admitted species branching order in the phylogenic tree, as captured by the phylogenies on X chromosomes, suggests that *An. arabiensis* would branch at this position (Fontaine et al. 2015; Thawornwattana et al. 2018; Müller et al. 2021). Therefore, it is plausible that this peculiar haplotype carried by AN0293_C_CMS sample could represent a historical relic haplotype of the original *An. arabiensis* mitogenomes (or from a closely related unsampled species) before being fully replaced by those of *An. gambiae* and/or *An. coluzzii*. This haplotype may still be segregating at low frequency in the gene pool of the three species. The ongoing phase-3 of The Anopheles gambiae 1000 genomes consortium (2021) now includes hundreds of samples from *An. arabiensis*. This project will provide further insights on this topic, and whether or not this haplotype, or closely related ones, are still segregating in the mtDNA gene pool of *An. arabiensis*.

**Fig. 3.**
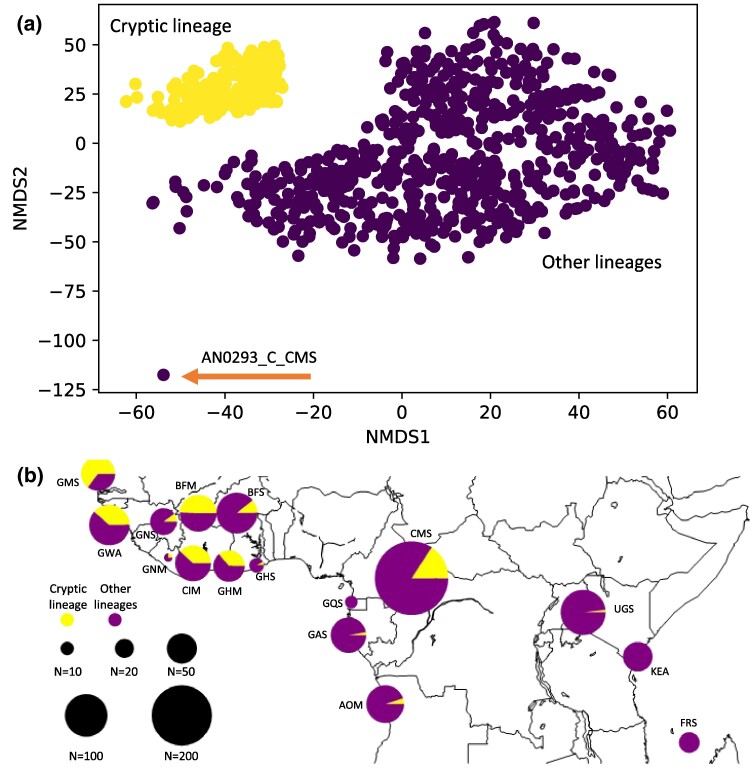
Genetic distance and geographic distribution of the cryptic lineage versus the others. a) Distance-based nonmetric multidimensional scaling (nMDS) analysis of the 1,142 mtDNA sequences from *The Ag1000G Consortium* with a color-coding based on a hierarchical clustering analysis. The cryptic lineage is in yellow. The outlier sample at the bottom (AN0293_C_CMS) is most likely a relic of *An. arabiensis* haplotype (see Fig. 2). b) Geographic distribution of the cryptic lineage versus the others (see also supplementary fig. S7, Supplementary Material online for distributions of the *π_XY_* and *D_XY_* distances within and between haplogroups).

While most of the mtDNA haplotypes carried by *An. gambiae*, *An. coluzzii*, and *An. arabiensis* were closely related, as shown by the short branches on the phylogenetic tree (Fig. 2 and supplementary figs. S5 and S7, Supplementary Material online) and on the distance-based nonmetric multidimensional scaling (nMDS) (Fig. 3 and supplementary fig. S6, Supplementary Material online), a group of 244 samples (232 from the The Anopheles gambiae 1000 Genomes Consortium (2020) and 12 from Fontaine et al. (2015)) clustered into a distinctive clade (hereafter called the “cryptic lineage”) (Figs. 2 and 3 and supplementary figs. S5 to S7, Supplementary Material online). This cryptic lineage included 199 distinct haplotypes (Table 1) and displayed a higher level of divergence than the others within the mtDNA gene pool of *An. gambiae*, *An. coluzzii*, and *An. arabiensis*, yet similar-to-slightly-lower than for the clades containing the other four species of the AGC. The branch length of the cryptic lineage on the phylogenetic tree was indeed larger than the others in the phylogenetic tree, and intermediate compared with the other species of the AGC (Fig. 2 and supplementary figs. S5 and S7a, Supplementary Material online). This was also shown by the distributions of the genetic distances within and between lineages (supplementary fig. S7b, Supplementary Material online), as well as the departure of the cryptic lineage from the others on the distance-based nMDS (Fig. 3a and supplementary fig. S6, Supplementary Material online). Interestingly, the geographic distribution of this cryptic group matched closely with the geographic distribution of *An. coluzzii*, mostly prevalent in the African “*far-west*” side of the distribution of the two species, and decreasing in frequency eastwards and southwards (Fig. 3b). The prevalence of the cryptic lineage was the most important in the hybrids (taxonomically uncertain) populations where it reached *ca.* 50% of the samples (up to 65% for the populations of the Gambia (GMS) and 40% of the Guinea-Bissau (GWA)), then composing 31% of the *An. coluzzii* samples, and less than 10% of the *An. gambiae* samples (Table 1, Fig. 3b, and supplementary fig. S6, Supplementary Material online). This clear enrichment of the cryptic mtDNA lineage in the populations from the African *far-west*, especially in the hybrid and *An. coluzzii* populations, its level of divergence compared with the other mtDNA lineages which was comparable to the levels observed among species of the AGC, yet slightly smaller (Fig. 2 and supplementary figs. S5 and S7, Supplementary Material online), together with the West-to-East gradual decline, all these observations suggest that it could be related to the species isolation between *An. gambiae* and *An. coluzzii*. Distributions of the nucleotide diversity along the mitogenome of this cryptic lineage and the others were similar (supplementary fig. S8, Supplementary Material online), and so were Tajima's *D* values (Table 1). These results suggest that this cryptic lineage is not simply the result of a recent selective sweep. Furthermore, the average number of nucleotide differences (*D_xy_*) between this cryptic lineage and the others sampled among *An. gambiae*, *An. coluzzii*, and *An. arabiensis* (*D_xy_* = 70 for the entire mitogenome sequence or 4.7 × 10^−3^ per site) was larger than the pairwise distances within the cryptic and the other lineages (*π_xy_ =* 2.2 × 10^−3^ and 3.6 × 10^−3^ per site, respectively); yet the values were halfway with the distances observed when comparing with the other species of the AGC (*An. melas*, *An. merus*, *An. quadriannulatus*, and *An. bwambae*) with a *D_xy_* = 10.1 × 10^−3^ per site (supplementary fig. S7, Supplementary Material online). Assuming a clock-wise neutral molecular evolution of the mitogenome (Gillespie and Langley 1979), a mutation rate ranging between 10^−7^ and 10^−8^ per site and per generation (as estimated in *Drosophila melanogaster*) (Haag-Liautard et al. 2008), and roughly ten generations per year, the genetic distance we observed between the cryptic lineage and the others would approach roughly the split time between *An. gambiae* and *An. coluzzii* estimated between 40 × 10^3^ and 60 × 10^3^ years before present (The Anopheles gambiae 1000 Genomes Consortium 2017; Thawornwattana et al. 2018; Müller et al. 2021). All these arguments support the hypothesis that this cryptic lineage may be a phylogeographic legacy of the split between the two sister species, although we cannot fully rule out other plausible origins as well (for example selection; see below).

### Significant mtDNA Genetic Structure Among and Within *An. gambiae* and *An. coluzzii*

An analysis of molecular variance (AMOVA) (Excoffier et al. 1992) showed that most of the mtDNA variation was distributed within populations (87.0%), but significant variance partitioning was also observed between populations (9.6%, *P* < 0.001) and between species as well (3.4%, *P* < 0.007) (Table 2). Level of population differentiation (supplementary fig. S9, Supplementary Material online) expressed as *F_ST_* values among populations between nuclear genome (nuDNA) from The Anopheles gambiae 1000 Genomes Consortium (2020) and mtDNA genome were strongly correlated (supplementary fig. S10, Supplementary Material online). *F_ST_* values at the nuclear genome explained *ca.* 80% of the mtDNA *F_ST_* values (*P* < 0.001). All comparisons involving the isolated island population of Mayotte (FRS) displayed both high values at the nuDNA and mtDNA genome, the highest mtDNA values being observed between the island populations of Mayotte (FRS) and Bioko (GQS) (supplementary figs. S9 and S10, Supplementary Material online). Such elevated levels of mtDNA and nuDNA differentiation reflect the small long-term effective population size and limited gene flow, with potential repeated bottleneck/founder effects. All these contribute to a strong genetic drift of this Mayotte Island populations (FRS), as previously reported (The Anopheles gambiae 1000 Genomes Consortium 2020). Globally, genetic differentiation observed at the mtDNA were overall higher than those at the nuclear genome, which likely reflect the reduced effective size of the mtDNA compared with the nuDNA. Exceptions included all comparisons involving the taxonomically uncertain and very peculiar population of Kenya (KEA), where the *F_ST_* values were lower or equivalent (supplementary figs. S9 and S10, Supplementary Material online). Overall, we observed a high concordance between the mtDNA and nuDNA levels of population differentiation. These results further underline that both geographical location and, to a lesser extent, species differentiations within and between *An. gambiae* and *An. coluzzii* are major determinants of mtDNA variation.

**Table 2:** AMOVA describing the variance partitioning at three hierarchical levels: between species, between populations within species, and within populations

	df	SSD	MSD	Var (Sigma)	Variation (%)	*ɸ*	*P*-value
*ɸ*_CT_ (between species)	1	0.049	0.049	6.96 × 10^−05^	3.4	0.03	<0.007
*ɸ*_SC_ (among populations within species)	11	0.154	0.014	1.94 × 10^−04^	9.6	0.10	<0.001
*ɸ*_ST_ (within populations)	925	1.627	0.002	1.76 × 10^−03^	87.0	0.13	<0.001
Total	937	1.829	0.002	2.02 × 10^−03^	100	–	–

The AMOVA was conducted with the TN93 + gamma model of sequence evolution. The analysis was conducted considering only populations from the Ag1000G that were taxonomically unambiguous (*n* = 938, see Fig. 1), thus removing the hybrid taxonomically uncertain populations from The Gambiae (GMS) and Guinea-Bissau (GWA), as well as the population from Kenya (KEA). The table provides the main results of the AMOVA including the degree of freedom (df) at each level, the sum square and mean square deviations (SSD and MSD), variance component (σ), and variance proportion (%), *ɸ*-statistics, and *P-*value from 1,000 permutation test.

### MtDNA Isolation-by-Distance Patterns Reflect Distinct Life Histories Between *An. gambiae* and *An. coluzzii*

Previous studies showed that genetic differentiation (i.e. *F_ST_* or its linearized equivalent *F_ST_*/(1−*F_ST_*)) at the nuclear genome significantly increased with geographic distance in *An. gambiae* and *An. coluzzii* (Lehmann et al. 2003; The Anopheles gambiae 1000 Genomes Consortium 2020). This isolation-by-distance (IBD) pattern was significantly stronger in *An. coluzzii* than in *An. gambiae*, translating into reduced local effective population size and/or reduced intergenerational dispersal distance in the first compared with the second species (see Fig. 3 in The Anopheles gambiae 1000 Genomes Consortium 2020). In line with these findings at the nuclear genome, we found significant IBD at the mtDNA as well when considering all populations irrespective of the species (Mantel's *r =* 0.35; *P* < 0.003; *n* = 13), with a very strong signal among populations of *An. coluzzii* (Mantel's *r =* 0.96; *P* = 0.017; *n* = 5), and a weaker marginal signal among populations of *An. gambiae* (Mantel's *r =* 0.32; *P* = 0.095; *n* = 8) (supplementary table S4, Supplementary Material online). However, these analyses included populations that were found genetically isolated by geographic barrier to geneflow when analyzing the nuclear genome (Angola—AOM in *An. coluzzii*; Gabon—GAS; and Mayotte Island—FRS in *An. gambiae*) (The Anopheles gambiae 1000 Genomes Consortium 2020). These geographic barriers to dispersal can artificially inflate the IBD patterns without necessarily implying reduced neighborhood size, which is the product of reduced local effective population density and intergenerational dispersal distance that increase local genetic drift (Wright 1946; Rousset 1997). The IBD signal among populations within species becomes weaker and not statistically different from zero when removing geographically isolated populations (AOM, GAS, or FRS) from the IBD analysis (*An. coluzzii* Mantel's *r* = 0.46, *P* = 0.167, *n* = 4; *An. gambiae* Mantle's *r* = −0.18; *P* = 0.617; *n* = 6). Nevertheless, despite the lack of significant results likely due to the small number of sampled populations, the strength of association between genetic and geographic distances still remain strong and positive in *An. coluzzii* with a *r^2^* value of 0.21, which is very comparable to the *r^2^* value of 0.22 observed at the nuclear genome (see Fig. 3b in The Anopheles gambiae 1000 Genomes Consortium 2020). This contrasts with the lack of any detectable IBD signal at the mtDNA genome among populations of *An. gambiae* and the very weak IBD signal found on the nuclear genome. These results are consistent with the distinct life history and dispersal strategies between the two species (Dao et al. 2014; Huestis et al. 2019; Hemming-Schroeder et al. 2020; Faiman et al. 2022). A significant fraction of the populations of *An. coluzzii* from NW Africa endure locally the dry season by engaging into aestivation strategy to rebound from local founders when the wet season starts. In contrast, *An. gambiae* populations go locally extinct during the dry season and rebound after a certain lag time by long-distance migration. Since female mosquitoes potentially disperse more and live also longer than males (Yaro et al. 2022), we may have expected weaker evidence of IBD at the mtDNA compared with the signal found at the nuclear genome. However, we did not observe this effect. Thus, if this effect exists, it would be likely counter balanced by the strong differences in aestivation and dispersal strategies between the two species.

### MtDNA Variation in Line With Population Demography of *An. gambiae* and *An. coluzzii*, but With an Imprint of the Cryptic Lineage History

**Fig. 4.**
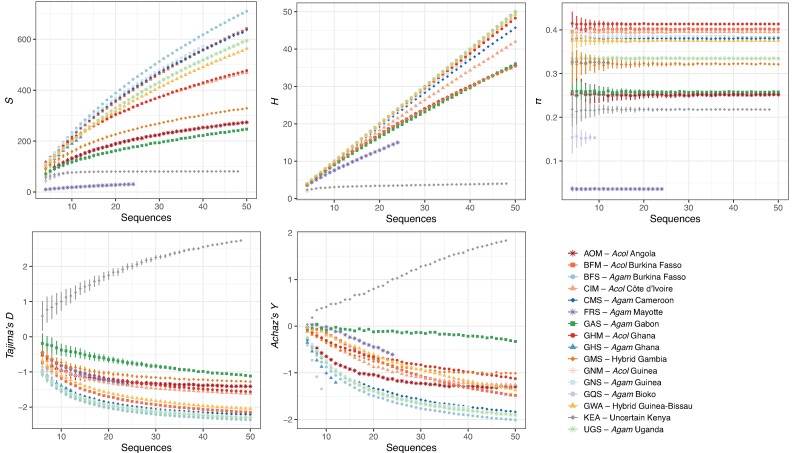
Mitochondrial genetic diversity statistics for each population of the Ag1000G. The statistics shown include the number of segregating site (*S*), the number of haplotypes (*H*), the nucleotide diversity (*π*), Tajima's *D*, and Achaz's *Y*. The rarefaction curves describe the impact of varying sample size on the estimated values for each statistic and for each population. The mean and standard error values are reported for each sample size increment from 3 to 50.

Patterns of mtDNA variation among populations of *An. gambiae* and *An. coluzzii* (Table 1 and Fig. 4) were consistent with those previously reported at the nuclear genome (The Anopheles gambiae 1000 Genomes Consortium 2020). The exceptional genetic diversity previously observed at the nuclear genome also manifested at the mtDNA level by a high overall level of haplotype diversity (*HD* = 0.999), with 910 distinct haplotypes found in 1,142 samples, an average number (*K*) of 58 nucleotide differences between pairs of haplotypes, and 3,017 segregating sites including one third of singletons (Table 1).

Rarefaction curves, which account for differences in population sample sizes, for the number of segregating sites (*S*) and the number of haplotypes (*H*) kept increasing with the sample size in most populations. In other words, as more samples are being added, the detection of rare variants (especially singletons) increases and the value of *S* and *H* as well. These curves clearly showed that the plateau was not within reach with a sampling up to 50, especially for the populations located North of the Congo River Basin and West to the Rift Valley (Fig. 4). In terms of nucleotide diversity (*π*) (which is not sensitive to sample size, but its variance is), these populations were also among the most diversified, with the highest values observed for the *An. coluzzii* populations from the NW Africa, followed by the *An. gambiae* populations from the same regions, and the hybrid (taxonomically uncertain) population in the Guinea-Bissau (GWA). These high levels of nucleotide diversity (*π*) actually reflected populations in which there was a mixed proportion of haplotype from the cryptic and other mito-groups identified in the phylogenetic analyses (Fig. 2 and supplementary figs. S5 and S7, Supplementary Material online). Values of nucleotide diversity (*π*) decreased in populations where the haplotype mixture between cryptic and other common lineages decreases, for example in the hybrid (taxonomically uncertain) population of Gambia (GMS) where the cryptic lineage dominates or in the *An. gambiae* population of Uganda (UGS) where it is almost absent. Overall, the high level of mtDNA variation combined with very negative values for Tajima's *D* or Achaz's *Y* statistic (Achaz 2008) indicate an excess of rare variants. These results support previous demographic inference modeling, showing large effective population sizes in the NW Africa distribution ranges of the two species and evidence for historical population expansions (The Anopheles gambiae 1000 Genomes Consortium 2017, 2020). These conditions where genetic drift is very ineffective are favorable to maintain high genetic diversity.

The (semi-)isolated populations from Gabon (*An. gambiae—*GAS) and Angola (*An. coluzzii*—AOM) displayed intermediate values of genetic diversity and Tajima's *D* and Achaz's *Y* values closer to zero (Table 1 and Fig. 4). This is consistent with a historically more stable population size, and reduced effective size as previously reported (The Anopheles gambiae 1000 Genomes Consortium 2017, 2020; Daron et al. 2024). The two *An. gambiae* populations from the islands of Mayotte (FRS) and Bioko (GQS) also departed from the other populations at the mtDNA variation with very low nucleotide and HD, and slightly negative Tajima's *D* and Achaz's *Y* values. These are further evidence for small effective population size, and suggestive of strong bottlenecks (or founder effects). In these island populations, the number of haplotypes was small and closely related to each other, with excess of rare variants, as expected after strong bottlenecks which can result from cyclic variation in population sizes, with possibly repeated founder events.

The taxonomically uncertain population from Kenyan (KEA) was already known for its very peculiar patterns of genetic diversity at the nuclear genome, with a genomic profile close to a colony population with mixed ancestry from *An. gambiae* and *An. coluzzii* (see Fig. 4 in The Anopheles gambiae 1000 Genomes Consortium 2020). The Kenyan population was also an outlier population at the mtDNA genome with only four distinct haplotypes detected that differ from each other at only ∼32 sites with almost no singletons, thus a very low haplotype diversity (HD = 0.65) compared with the other populations, and the only population in the Ag1000G sampling with highly positive Tajima's *D* and Achaz's *Y* values (Table 1 and Fig. 4). These highly positive values are expected for incomplete bottlenecks and/or admixture of diverged haplotypes (multiple recent bottlenecks-founder effects), whereby alleles are segregating at intermediate frequency.

### The Cryptic mtDNA Lineage: A Phylogeographic Legacy of the Split Between *An. gambiae* and *An. coluzzii*

We investigated further the specificities of the distinctive cryptic mtDNA lineage (Fig. 2 and supplementary figs. S5 and S7, Supplementary Material online) to better understand its potential evolutionary origin(s). We tested whether its occurrence was associated with the potential occurrence of *Wolbachia* infection, the population genetic structure at the nuclear genome, as estimated using a principal component analysis (PCA) following The Anopheles gambiae 1000 Genomes Consortium (2017, 2020), and other genomic features previously characterized for these samples, including major chromosomal inversions (2L^a^, 2R^b,c,d,u^), and insecticide resistance mutations (*rdl226*, *vgsc995*) (supplementary table S2, Supplementary Material online).

The intracellular and intraovarian *Wolbachia* bacterium is frequently found in insects and can be a strong manipulator of insect reproductive biology, impacting physiology, behavior, creating CIs, and could even act as a speciation agent (Rokas 2000; Werren et al. 2008; Galtier et al. 2009; Dong et al. 2021; Bruzzese et al. 2022 ; Dowling and Wolff 2023). *Wolbachia* could thus have significant impacts on mitochondrial heritability and its genetic variation. It was previously detected in *An. gambiae* and *An. coluzzii*, even though the vertical transmission or impacts on the reproductive biology of these mosquitoes is still debated (Baldini et al. 2014; Shaw et al. 2016; Gomes et al. 2017; Gomes and Barillas-Mury 2018; Jeffries et al. 2018, 2021; Pascar and Chandler 2018; Ayala et al. 2019; Chrostek et al. 2019; Straub et al. 2020; Bamou et al. 2021).

We used the method of Pascar and Chandler (2018) to detect *Wolbachia* occurrence, using the unmapped Illumina short-read data of The Anopheles gambiae 1000 Genomes Consortium (2020). Using a lenient set of filters (at least three reads mapping to *Wolbachia* sequence with at least 90 bp and 90% sequence identity), we found 111 (9.7%) individual mosquitoes carrying reads blasting to the *Wolbachia* supergroup A (supplementary fig. S11 and table S5, Supplementary Material online). This detection rate dropped to 27 (2.4%) positive individuals when using stricter detection filters (three reads blasting to Wolbachia sequences with at least 98 bp length and 95% identity) similar to those used by Pascar and Chandler (2018). *Wolbachia* was primarily detected in the *An. gambiae* population of Mayotte (FRS; lenient: 83% or strict: 17%), and in the *An. coluzzii* populations of Côte d’Ivoire (CIM; lenient: 55% or strict: 23%) and Ghana (GHM; lenient: 44% or strict: 4%) (supplementary fig. S11 and table S5, Supplementary Material online). These infection rates were quite low, especially if we consider the stricter criteria of Pascar and Chandler (2018). These rates were in line with previous reports by Chrostek et al. (2019) who even questioned the natural occurrence of *Wolbachia* in natural populations of *An. gambiae* and *An. coluzzii.* Chrostek et al. (2019) argued that such a low number of reads could come from ingested food, or mosquito parasites infected by *Wolbachia* (e.g. nematodes). We did not find any significant association between the *Wolbachia* potential occurrence and the cryptic mtDNA lineage (ranked predictive power of cross-features *x2y* metric = 0; supplementary fig. S12, Supplementary Material online).

Population genetic structure was estimated by a PCA on 100k independent single nucleotide polymorphisms (SNPs) from the nuclear genome (supplementary fig. S13, Supplementary Material online), following the same procedure as in The Anopheles gambiae 1000 Genomes Consortium (2017, 2020). The PC1, PC6, and (to a lesser extent) PC2 were significant predictors of the cryptic mtDNA lineage occurrence, explaining between 13% and 15% of the cryptic mtDNA lineage variation for PC1, 21% for PC6, and 2% for PC2 (supplementary fig. S12, Supplementary Material online). PC1 discriminates *An. coluzzii* from *An. gambiae*, PC6 splits the hybrid (taxonomically uncertain) populations from the other populations of *An. coluzzii* and *An. gambiae*, and PC2 reflects the strong differentiation of the Angolan (AOM) population from the other *An. coluzzii* and *An. gambiae* populations (supplementary fig. S13, Supplementary Material online). Altogether, these associations between the PCs and the cryptic mtDNA lineage occurrence underline its variation according to species and geography visually displayed in Fig. 3b. Beside the PCs, no other genomic features tested here significantly correlated with the occurrence of the cryptic mtDNA lineage variation in natural populations, except for the 2La chromosomal inversion frequency. However, the 2La inversion was also strongly associated with the population genetic structure capture by the PCs, suggesting its association with the cryptic mtDNA lineage could be an “echo” of the population genetic structure (supplementary fig. S12, Supplementary Material online).

Taken together, the above results add to the other arguments regarding its genetic diversity, its divergence compared with the other lineages, and its putative divergence time in line with the *gambiae-coluzzii* species split time; all of them suggests that the cryptic mtDNA lineage is likely a phylogeographic legacy of the split between *An. colluzzii* and *An. gambiae*. It likely arose during a period of isolation in *An. coluzzii*, as suggested by its level of divergence similar to the interspecific mtDNA divergence observed between species of the AGC, and by the enrichment of this lineage in the populations of *An. coluzzii* and in the taxonomically uncertain populations from the African *far-west* (Figs. 2 and 3a and supplementary figs. S5 and S7, Supplementary Material online). Together with the West-to-East gradient decline matching closely the distribution range of *An. coluzzii* (Fig. 3b), these results suggest that the two sister species went back into contact with an incomplete homogenization of the mtDNA gene pool.

### Mitonuclear Interactions Suggest Selection on the Mito-group Divergence Related to Metabolic Resistance to Pathogens and Insecticides

Selection may not have been initially involved in the split of the cryptic lineage, but it may have been implicated to some extent to prevent a full homogenization of the mtDNA gene pool(s) between *An. coluzzii*, *An. gambiae*, and the hybrid (taxonomically uncertain) populations, with possible mitonuclear interactions. To test this hypothesis, we conducted a genome-wide association study (GWAS), testing which SNPs on the nuclear genome were significantly associated with the cryptic mtDNA lineage occurrence (which is considered here as a binary variable). This GWAS analyses can be seen as a sophisticated way of testing linkage disequilibrium (LD) between the occurrence of the cryptic lineage and SNPs of the nuclear genome, while accounting for covariates. Among them, we used the six first PCs (supplementary fig. S13, Supplementary Material online) to account for population genetic structure, as well as *Wolbachia* occurrence (supplementary fig. S11 and table S5, Supplementary Material online), and the sex of the mosquitoes. As such GWAS analysis requires unrelated samples (Uffelmann et al. 2021), we excluded closely related sample pairs in the Ag1000G dataset with kinship coefficient exceeding the level of 2nd degree relatives (supplementary table S6, Supplementary Material online). The KING-robust method (Manichaikul et al. 2010), which relaxes the assumption of genetic homogeneity within population, identified multiple related pairs of samples within populations equal or exceeding the level of 2nd degree relatives, with some cases of full-sib or parent-offspring's, and even rare cases of monozygotic twins between pairs of mosquitoes (see supplementary figs. S14 and S15 and tables S7 and S8, Supplementary Material online). Full-siblings or parent-offspring's relationships in mosquitoes can occur if samples originated from larvae from a single female for example. Monozygotic twin's relationship can either reflect sample duplicates in the dataset or highly inbred samples as would be observed in samples coming from a laboratory colony. Unsurprisingly, the most impacted population was the Kenyan (KEA) one. Its peculiar genetic make-up is similar to a laboratory colony, as was previously spotted in The Anopheles gambiae 1000 Genomes Consortium (2017). However, instances of full-siblings and monozygotic twins were found in the populations from Cameroon (CMS) and Angola (AOM) (see supplementary figs. S14 and S15 and tables S7 and S8, Supplementary Material online). Overall, removing 98 samples from the dataset (supplementary table S9, Supplementary Material online) resolved all the issues allowing only up to the 3rd degree relative association between sample pairs. The cleaned SNPs dataset used in the GWAS included 1,044 unrelated samples (supplementary fig. S13b, Supplementary Material online) and 7,858,575 nuclear biallelic SNPs (supplementary table S10, Supplementary Material online). After removing related samples, and accounting for population structure, sex, and *Wolbachia* occurrence as covariates, the quantile-to-quantile plot and the genomic inflation factor (Lambda) were close to 1, indicating that the genomic control of the GWAS was adequate (supplementary fig. S16, Supplementary Material online).

**Fig. 5.**
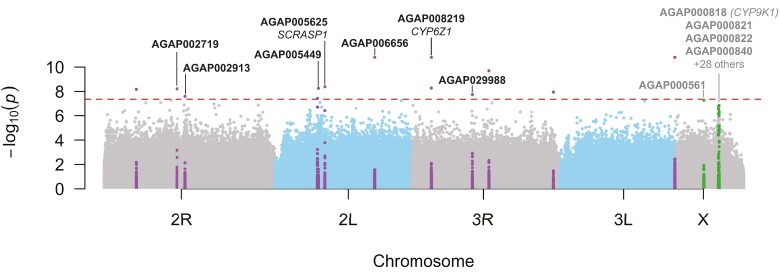
Manhattan plot showing the genetic associations between the cryptic mtDNA lineage (vs. the others) and each of the SNPs on the nuclear genome. The GWAS was conducted accounting for population structure using the six first PCs, sex, and *Wolbachia* occurrence as covariates. Each chromosome arms are colored-coded. The red dash horizontal line shows the Bonferroni-corrected significance threshold of 4.4×10^−8^. The 14 significant SNPs together with the SNPs 1 kb upstream or downstream are marked in purple. SNPs in green are those marginally significant on the X chromosome forming a clear “skyscraper”. Gene-ID and gene name, when an annotated transcript was available, are displayed. See supplementary fig. S16, Supplementary Material online for QQ plot, supplementary fig. S17, Supplementary Material online for a zoomed view of each significant SNPs, and supplementary fig. S18, Supplementary Material online for a zoomed view on the X chromosome.

The GWAS analysis identified 14 SNPs significantly associated with the cryptic mtDNA lineage occurrence with *P*-values lower than the Bonferroni adjusted threshold of 4.4 × 10^−8^ (Fig. 5 and supplementary fig. S17 and table S11, Supplementary Material online). Out of the 14 SNPs, 7 were found close (within 1 kb) or within transcripts, and 2 among them felt within 2 annotated genes: *SCRASP1 (AGAP005625)* and *CYP6Z1 (AGAP008219).* The gene encoding for the scavenger receptor *SCRASP1* was previously identified in *An. gambiae* as a prominent component involved in immunity response to *Plasmodium* infection, but also other pathogens like bacteria (Danielli et al. 2000; Christophides et al. 2002; Stathopoulos et al. 2014; Smith et al. 2016). By silencing this gene, Smith et al. (2016) showed that it was an important modulator of *Plasmodium* development in *An. gambiae*. These authors observed that *SCRASP1* was highly enriched after blood-feeding alone and speculated that it may contribute to a metabolic preemptive immune response activated by the hormonal changes that accompany blood feeding. Its role as cell surface receptors suggest that it may act as immuno-suppressors that when silenced, increase innate immune signaling in mosquito hemocyte populations.

The second genes significantly associated with the cryptic mtDNA haplogroup occurrence was *CYP6Z1*, encoding for a cytochrome P450 capable of metabolizing insecticide like the DDT in *An. gambiae* (Chiu et al. 2008). *CYP6Z1* is considered more generally as important insecticide resistance gene (Liu 2015; Ibrahim et al. 2016).

We also identified two additional suggestive mitonuclear association signals of interest on the X chromosome, with a marginal *P*-value ranging between 5.3×10^−8^ and 1.4×10^−7^ (Fig. 5 and supplementary fig. S18, Supplementary Material online). Only 27 other SNPs were found on the autosomes with similar *P-*values, indicating very low cherry-picking risk (Pavlidis et al. 2012). The first marginally significant signal on the X chromosome (AGAP000561) is located at *ca.* 9.95 Mb and encodes for a Piwi-interacting RNA (piRNA) previously identified as part of “reproductive and development” cluster involved in germline development and maintenance, spermatid development, oogenesis, and embryogenesis of *An. gambiae* (George et al. 2015). The authors suggested that these piRNA plays a significant role in the epigenetic regulation of the reproductive processes in *An. gambiae*. AGAP000561 is an ortholog of the *D. melanogaster* kinesin heavy chain (FBgn0001308), which plays a role in *oskar* mRNA localization to the pole plasm (Brendza et al. 2000).

The second mitonuclear marginal association signal of interest on the X chromosome was a clear “skyscraper” located between 15.24 and 15.78 Mb (Fig. 5). Zooming into this region revealed that the signal contained two skyscrapers with the highest association signals that are nested within a broader region with a distinctive elevation of the *P*-values (see supplementary fig. S18, Supplementary Material online). This distinctive region is not only of special interests for being marginally associated with the cryptic mito-group split, but it was also identified in many populations of *An. gambiae* and *An. coluzzii* with strong signal of recent positive selection and association with metabolic insecticide resistance involving also the mitochondrial oxidative phosphorylation (OXPHOS) respiratory chain (The Anopheles gambiae 1000 Genomes Consortium 2017; Ingham et al. 2021b; Lucas et al. 2023) (see also the *Ag1000G Selection Atlas*; https://malariagen.github.io/agam-selection-atlas/0.1-alpha3/index.html*).* A total of 32 genes overlaps with this focal region (supplementary fig. S18, Supplementary Material online). Among them is the well-known cytochrome p450 encoded by *CYP9K1*, an important metabolic insecticide resistance gene (Main et al. 2015; Vontas et al. 2018; Lucas et al. 2023). Even if that gene is within the elevated *P*-value region, it is located 62 kb upstream from the first skyscraper signal. The first highest signal overlapped with AGAP000820 (CPR125—cuticular protein RR-2 family 125), AGAP00822 and AGAP00823 (CD81 antigen). The second skyscraper was centered close to AGAP000840 (amiloride-sensitive sodium channel) and to AGAP000842 (NADH dehydrogenase (ubiquinone). Other noteworthy genes in that genomic region included include AGAP000849 (NADH dehydrogenase (ubiquinone) 1 beta subcomplex 1) and AGAP0008511 (cytochrome c oxidase subunit 6a, mitochondria). These results thus suggest that the mtDNA lineage haplogroups are associated with mitochondrial genes located in the nuclear genome, as well as genes involved directly or indirectly in insecticide resistances mechanisms (cytochrome p450 and also cuticular regulation genes) and immunity.

Overall, these results support the hypothesis of a tight coevolutionary history between the two genomic compartments and suggest that these mitonuclear interactions may have left imprints on the mtDNA genetic variation. The associations between the mtDNA lineages with genes involved in metabolic resistance to pathogens (*Plasmodium* and bacteria) and insecticides support the emerging picture of the key role played by mitochondria, and especially the OXSPHOS pathway in mosquito immunity and insecticide resistance. Previous studies demonstrated that mitochondrial reactive oxygen species (mtROS) produced by the OXSPHOS pathway modulate *An. gambiae* immunity against bacteria and *Plasmodium* (Molina-Cruz et al. 2008). Ingham et al. (2021a) were already discussing the disruption of parasite development due to changes in redox state shown experimentally through reducing catalase activity which in turn reduces oocyst density in the midgut (Molina-Cruz et al. 2008), while the initial immune response to parasite invasion consists in a strong mtROS burst (Molina-Cruz et al. 2008; Castillo et al. 2017).

Evidence implicating the mitochondrial respiration, OXPHOS pathway, and more generally, the mosquito metabolism into metabolic insecticide resistance is increasingly reported in the literature (e.g. Oliver and Brooke 2016; Ingham et al. 2021a, 2021b; Lucas et al. 2023). Ingham et al. (2021b) used a multi-omics study to investigate the causative factors involved in the reestablishment of pyrethroid resistance in a population of *An. coluzzii* colony from Burkina Faso after a sudden loss of the insecticide resistance. Beside the involvement of the 2Rb inversion and of the microbiome composition, the authors detected an increase in the genes expression within the OXPHOS pathway in both resistant populations compared with the susceptible control, which translated phenotypically into an increased respiratory rate and a reduced body size for resistant mosquitoes. This, and previous studies (Oliver and Brooke 2016; Ingham et al. 2017, 2021a), clearly indicated that elevated metabolism was linked directly with pyrethroid insecticide resistance. Additionally, Lucas et al. (2023) investigated novel loci associated with pyrethroid and organophosphate resistance in *An. gambiae* and *An. coluzzii* using a GWAS, which also implicated the involvement of a wide range of cytochrome p450, mitochondrial, and immunity genes (including also the same genomic region on the X chromosome as the one we detected here). Both Ingham et al. (2021b) and Lucas et al. (2023) further pointed out possible cross-resistance mechanisms in metabolic insecticide resistance at large, in which the mosquito metabolism, mitochondrial respiration, the OXPHOS pathway, and mtROS production, all seem to play an important role.

## Conclusions

In this study, we showed that the determinants of mitochondrial genetic variation are multifarious and complex. In agreement with previous studies (Fontaine et al. 2015; Thawornwattana et al. 2018; Müller et al. 2021), the mtDNA phylogeny clearly illustrated the previously reported highly reticulated evolutionary history of the AGC. On the one side, the three most widely distributed species—*An. gambiae*, *An. coluzzii*, and *An. arabiensis*—form a rather homogeneous mtDNA gene pool clearly illustrating the extensive level of introgression that occurred between them over the evolutionary timescale of the AGC. On the other side, other species of the AGC cluster in a well-diverged monophyletic clade, where each species forms a clearly distinct monophyletic group. One haplotype in the mtDNA gene pool of *An. gambiae*/*An. coluzzii* clustered close to *An. quadriannulatus*, in a position of the species tree where *An. arabiensis* was placed according to the species informative loci on the X chromosome and the autosomes (Fontaine et al. 2015). This may suggest that a mito-lineage belonging to the *An. arabiensis* ancestral pool (or that from a closely related species) might still be segregating in this joined mtDNA gene pool of the three most widespread species in the AGC.

Mitochondrial introgression was also detected in other species, notably between *An. bwambae* and most likely *An. gambiae*. The mitochondrial phylogenetic clustering of all the saltwater-tolerant members of the AGC (*An. merus*, *An. melas*, and *An. bwambae)* into a strongly supported monophyletic group also departed from the admitted species branching order (Fontaine et al. 2015; Thawornwattana et al. 2018; Barrón et al. 2019). This suggests that historical mtDNA capture or selection from ancestral standing genetic variation may have occurred, possibly involving selective processes related to specialization to a very distinct salty larval habitat compared with the other freshwater-tolerant species of the AGC, and to the majority of the *Anophelinae* species (Bradley 1994, 2008). A proper population genetic study investigating this specialization from an evolutionary perspective still remains to be done.

Population structure, demography, and dispersal were found to be key drivers shaping the mtDNA variation across the African populations of *An. gambiae* and *An. coluzzii*. The patterns identified mostly followed those previously reported at the nuclear genomes (The Anopheles gambiae 1000 Genomes Consortium 2017, 2020). Despite the extensive level of gene flow between *An. gambiae* and *An. coluzzii*, significant variance partitioning between species was still detectable. Even more striking was a clearly distinct mito-lineage composed of 244 samples from *An. gambiae* and *An. coluzzii*. This lineage displayed a level of divergence significantly larger than the value observe among the other lineages segregating among the *An. gambiae*, *An. coluzzii*, and *An. arabiensis* mtDNA gene pool. Its divergence was comparable to, yet half than, the mtDNA divergence observed between the species of the AGC. Its distribution closely matched the distribution of *An. coluzzii* with a West-to-East and North-to-South decreasing frequency gradient and its divergence time approached the split time estimated between *An. gambiae* and *An. coluzzii*. All these arguments suggest that this cryptic lineage may be a phylogeographic legacy of the species isolation followed by a secondary contact between *An. gambiae* and *An. coluzzii* with incomplete homogenization. Its frequency was clearly associated with species divergence (being enriched in *An. coluzzii* compared with *An. gambiae*), mitochondrial level of diversity, and with population structure, but it was not linked with the rare *Wolbachia* occurrence detected from the short-read data. Once accounting for these variables in a GWAS-like study, we found significant associations between the cryptic lineage occurrence and SNPs of the nuclear genome mostly from genes involved in metabolic resistance to pathogens and insecticides. These results suggest that the phylogeographic split of mitochondrial lineages and its incomplete rehomogenization after the secondary contact may have involved selective processes and may imply a certain mitonuclear coevolution process between the two genome compartments. These associations support the picture emerging in the recent literature underlining the key role played by the respiratory metabolism, the OXPHOS pathway, and the generation of reactive oxygens in the metabolic resistance to pathogens and to insecticides.

Cross-resistance mechanisms are increasingly recognized as a major threat to vector control strategy allowing mosquitoes to adapt to insecticides (Ingham et al. 2021b; Lucas et al. 2023). Our results call for additional studies characterizing further the extent of mitonuclear associations, the role of mitochondria in adaptive processes to pathogens and insecticides, and a better understanding of the expected tight coordination and coevolution between the mitochondrial and nuclear genome. By integrating both mtDNA and nuDNA, this study underlines that the mtDNA locus, once considered as a nearly neutral locus and thus informative on the phylogenetic history of species, has in fact a much more complex evolution in *Anopheles* mosquitoes where all the evolutionary forces (drift, migration, mutation, and multiple type of selection) interact. Such integration of nuclear and mitogenomic study are still rare, but necessary to further our understanding of insect genomic evolution (Cameron 2014).

## Materials and Methods

### Sampling and Whole-Genome Short-Read Data

We retrieved whole-genome short-read (WG-SR) data (100 bp paired-end Illumina sequencing) from 74 mosquito specimens for six species of the AGC from Fontaine et al. (2015), including *An. gambiae* sensu stricto (s.s.), *An. coluzzii*, *An. arabiensis*, *An. quadriannulatus*, *An. melas*, and *An. merus*. As mtDNA genomes of these samples were previously assembled, we compared them with the ones produced using the new pipeline developed in the present study. We extracted reads that did not map to the nuclear reference genome and used them to assemble mitogenome. We included also WG-SR data from three specimens of a seventh species—*An. bwambae—*that were generated as part of the Anopheles 16 Genomes Project (Fontaine et al. 2015; Neafsey et al. 2015). See the complete sampling details in supplementary fig. S1 and table S1, Supplementary Material online. We also retrieved WG-SR data from The Anopheles gambiae 1000 Genomes Consortium (2020) phase-2 AR1 release consisting of 1,142 wild-caught mosquito specimens including *An. gambiae s.s.* (*n* = 720), *An. coluzzii* (*n* = 283), and hybrid (*n* = 139) from 16 geographical sites (Fig. 1 and supplementary table S2, Supplementary Material online).

Previously generated *An. gambiae* reference mitochondrial genome (GenBank ID: L20934.1) (Beard et al. 1993) was used to guide the assembly of the *AutoMitoG* pipeline. The mitochondrial sequences of *An. christyi* and *An. epiroticus* from Fontaine et al. (2015) were also included as outgroup sequences for phylogenetic analyses.

### Mitochondrial Genomes Assembly and Alignment

Information about the software versions is provided in supplementary table S3, Supplementary Material online. From the WG-SR files mapped to nuclear reference genomes (bam files) obtained from Fontaine et al. (2015) and The Anopheles gambiae 1000 Genomes Consortium (2020), we extracted reads that did not mapped to the nuclear reference genome and converted them to paired-reads fastq files using *Samtools* (Li et al. 2009) and *Picard* Tools (http://broadinstitute.github.io/picard/). We wrote the *AutoMitoG* (*Automatic Mitochondrial Genome assembly*) pipeline to streamline the mitochondrial genome assembly process (available at https://github.com/jorgeamaya/automatic_genome_assembly, supplementary fig. S2, Supplementary Material online). As a general overview, the pipeline starts by randomly sampling paired-reads from each file at a 5% rate. Then, the pipeline proceeds to assemble the mitogenome using a modified version of MITObim (Hahn et al. 2013) (see details below and in supplementary fig. S2, Supplementary Material online) and evaluate the quality of the assembly. This is done by counting the number of ambiguities outside the *D-loop* CR; a region prone to sequencing and assembly errors due to the AT-rich homopolymer sequences. If ambiguities remain in the mtDNA assembly, the previous steps are repeated iteratively, increasing the sampling rate of the paired-reads (fastq) file by 5% until the assembled mitogenomes show no ambiguities or until 100% of the reads are used. If ambiguities persist after reaching a sampling rate of 100%, the assembly with the least number of ambiguities is selected by default. Finally, assembled mitogenome sequences together with the previously assembled reference genome (Beard et al. 1993) and outgroup mtDNA sequences were aligned to each other with MUSCLE (Edgar 2004).

The *AutoMitoG* pipeline relies on a modified version of *MITObim* (Hahn et al. 2013) to assemble the mtDNA genomes. Subsampling of the paired-reads is performed to achieve two purposes: (1) minimize the number of ambiguous base calls—these can result from conflicting pairing of reads from mitochondrial origin with reads possibly originating from nuclear mitochondrial DNA copies (NUMTs), reads with sequencing errors, and reads that originated from possible contamination. Since the number of reads from mitochondrial origin is orders of magnitude larger in the WG-SR data than the number of reads from other sources, subsampling safely reduces offending reads; (2) to normalize the dataset coverage, which speeds up *MITObim* calculations, as the proportion of mitochondrial reads can differ between samples and studies. Indeed, *MITObim* performs best with sequencing depth between 100 and 120× for Illumina reads (Hahn et al. 2013).

*MITObim* performs a two-step assembly process (see Fig. 2 in Hahn et al. (2013)). First, it maps reads to a reference genome, here the mtDNA genome of *An. gambiae* from Beard et al. (1993), to generate a “backbone”; second, it extends this “backbone” with overlapping reads in an iterative de novo assembly procedure. Thanks to its hybrid assembly strategy, *MITObim* perform well even if the samples and the reference genome are phylogenetically distant (Hahn et al. 2013). We forced majority consensus for nonfully resolved calls during the backbone assembly and during the backbone iterative extension, for which we customized *MITObim*'s code. The original version of *MITObim* does not force majority consensus and was not used in this study. However, it is included as an option in the pipeline for the benefit of users who may prefer less stringent assembly criteria. See supplementary fig. S2, Supplementary Material online for further information on the pipeline usage and the corresponding documentation in the GitHub page.

We compare the newly assembled mitogenomes with those previously generated in Fontaine et al. (2015) (*n* = 74). For that purpose, we first aligned mitogenome sequences from the two studies, cropped out the CR (sequence length = 14,844 bp) following Fontaine et al. (2015) as it is prone to sequencing and assembly errors. Then, for each pair of mtDNA assemblies (new vs. previous) coming from each of the 74 samples in Fontaine et al. (2015), we counted the number of pairwise differences. We also visually compared assemblies generated with the two pipelines by building a distance-based neighbor-joining tree (HKY genetic distance model) (supplementary fig. S4, Supplementary Material online). These steps were conducted in Geneious Prime® (2023.0.1, Build 2022-2011-28 12:49).

### Mitogenome Genetic Diversity and Phylogenetic Relationships

As an initial assessment of the mtDNA alignment characteristics, we calculated various estimators of genetic diversity per species and per location including: the number of INDEL sites, segregating sites (*S*), average number of differences between pairs of sequences (*K*), number of haplotypes (*H*), haplotype diversity (HD), nucleotide diversity (*π*) (Nei 1987), and Theta-Watterson (Θ_W_) (Watterson 1975; Nei 1987). Departures from neutral model were estimated using Tajima's *D* (Tajima 1989) and Achaz's *Y* (Achaz 2008). These statistics were computed using the C-library *libdiversity* developed by G. Achaz (https://bioinfo.mnhn.fr/abi/people/achaz/cgi-bin/neutralitytst.c).

We estimated the phylogenetic relationships among mtDNA haplotypes using PhyML v.3.3 (Guindon et al. 2010), using a GTR mutation model. Branch and node supports were calculated using the fast likelihood-based (aLRT SH-like) method. The mitogenome sequences from *An. christy* and *An. epiroticus* were used as outgroups to root the trees. Multiple ML phylogenetic trees were built: one only considering the 74 sequences from the *An. gambiae* species complex for comparative purpose with previously published ML tree in Fontaine et al. (2015), including also the 3 *An. bwambae* samples; and another tree considering all the mitogenome sequences including the 77 mitogenome sequences combined with the 1,142 sequences of *An. gambiae* and *An. coluzzii* samples from The Anopheles gambiae 1000 Genomes Consortium (2020).

In order to provide an alternative visualization of the phylogenetic relationships given the large size of the total alignment, we also visualized mtDNA genetic variation among the 1,142 sequences of *An. gambiae* and *An. coluzzii* samples from The Anopheles gambiae 1000 Genomes Consortium (2020) into a reduced multidimensional space using a nMDS. For that purposed, we calculated a *p*-distance matrix among sequences using MEGA v.7 (Kumar et al. 2016) and performed the nMDS using the *ecodist* R-package (Goslee and Urban 2007). The nMDS results were further processed using *scikit*-learn v.0.22.1 (Pedregosa et al. 2011) to identify major clusters in the dataset, using a hierarchical clustering algorithm.

### MtDNA Genetic Structure in Natural Populations of *An. gambiae* and *An. coluzzii*

We first assessed how the mtDNA variation of 1,142 sequences of *An. gambiae* and *An. coluzzii* samples from The Anopheles gambiae 1000 Genomes Consortium (2020) partitioned among different levels of structuration using an AMOVA (Excoffier et al. 1992). We considered three nested hierarchical levels of stratification: between species, among populations within species, and within populations. The AMOVA was conducted with the TN93 + gamma model of sequence evolution using the *poppr R-package* (Kamvar et al. 2014) and the AMOVA function derived from the APE v5.6-4 R-package (Paradis et al. 2004; Paradis and Schliep 2019). Significance test was conducted using 1,000 permutations. The analysis was conducted considering only populations from the Ag1000G that were taxonomically unambiguous (*n* = 938, see Fig. 1), thus removing the hybrid/taxonomic uncertain populations from The Gambiae (GMS) and Guinea-Bissau (GWA), as well as the taxonomically uncertain population from Kenya (KEA).

Then, we quantified the level of mtDNA genetic differentiation among populations by calculating the pairwise *F_ST_* differences using Arlequin v3.5 and 1,000 permutations (Excoffier and Lischer 2010). We compared *F_ST_* values obtained for the mitochondrial DNA (mtDNA) with those previously reported for the nuclear genome (nuDNA) (The Anopheles gambiae 1000 Genomes Consortium 2020).

We characterized further the mtDNA variation, comparing genetic diversity estimators for each species at each locality. Since sample sizes vary among locations and can influence diversity estimators, we performed a rarefaction procedure to account for differences in sample sizes (Hurlbert 1971; Kalinowski 2004, 2005; Szpiech et al. 2008; Colwell et al. 2012; Ben Chehida et al. 2023). To do so, sequences from each location were randomly sampled incrementally, starting with three sequences up to a maximum of 50 sequences or until there were no more sequences available for the specific location. This random subsampling with replacement of the sequences was repeated 5,000 times for each sample size increment (from 3 to 50) to estimate the mean and standard error of the statistic of interest. This rarefaction analysis was applied for estimating the standardized number of segregating site (*S*), the number of haplotypes (*H*), the nucleotide diversity (*π*), Tajima's *D*, and Achaz's *Y* using python scripts and the *c-library libDiversity*. Results for each statistic were summarized as rarefaction curves.

IBD was computed following Rousset (1997). We derived the unbounded level of genetic differentiation *F_ST_*/(1−*F_ST_*) between pairs of populations and correlated the genetic distance with the geographic distance, expressed as the great circle distance (in log_10_ unit) globally across species, and also for each species separately. The strength and significance of the IBD was tested using a Mantel test implemented in *ade4* R-package (Dray and Dufour 2007) with 1,000 permutations of the geographic distance matrix. Since we were interested only in testing IBD within well-defined species, we removed the hybrid and taxonomically ambiguous populations (The Gambia—GM, Guinea-Bissau—GW, and Kenya—KEA) from this analysis. Likewise, we ran the analysis with and without the island *An. gambiae* population of Mayotte (FRS), as this population departs from the species' continuum (The Anopheles gambiae 1000 Genomes Consortium 2020).

### Detection of *Wolbachia* Infection in Natural Populations of the Ag1000G

MtDNA variation can be strongly impacted by cytoplasmic conflict with the endosymbiont *Wolbachia* (Galtier et al. 2009; Dong et al. 2021), and this latter has been reported in the AGC (Baldini et al. 2014; Shaw et al. 2016; Gomes et al. 2017; Gomes and Barillas-Mury 2018; Jeffries et al. 2018, 2021; Ayala et al. 2019; Chrostek et al. 2019; Straub et al. 2020). Therefore, we used the unmapped WG-SR data to diagnose the infection status of each mosquito specimen of *An. gambiae* and *An. coluzzii* from the Ag1000G phase-II (The Anopheles gambiae 1000 Genomes Consortium 2020). To that end, we screened the unmapped Ag1000G WG-SR data to detect *Wolbachia* specific sequences using MagicBlatst v.1.1.5 (NCBI) (Boratyn et al. 2019) following the procedure described in Pascar and Chandler (2018). WG-SR reads that did not map to the nuclear reference genome were compared with selected reference *wsp*, *ftsZ*, and *groE* operon sequences isolated from *Wolbachia* samples that are representative of supergroups A to D. For our analysis, we used the *Wolbachia* sequence database of Pascar and Chandler (2018), which includes 61 sequences of *Wolbachia* type A to D. We added four new sequences assembled by the authors to their database. These are *Wolbachia* sequences of type B also found in *An. gambiae* specimens (Pascar and Chandler 2018). Using the same *(strict)* detection criterion as in Pascar and Chandler (2018), a minimum of three reads with at least 98 bp length and 95% identity had to match with the same *Wolbachia* sequences for the specimen to be considered as infected. We also applied a more “*lenient*” criterion: a minimum of three reads with at least 90 bp length and 90% identity had to match with the same *Wolbachia* sequences for the specimen to be considered infected.

### Mitogenome Lineages Associations With Genomic Features and With SNPs of the Nuclear Genome

We explored the associations between the cryptic mitochondrial phylogenetic lineages (vs. other lineages) and the genetic variation on the nuclear genome using the genome-wide SNP data from The Anopheles gambiae 1000 Genomes Consortium (2020). We also considered the association of the mtDNA lineages with other covariates including population structure, *Wolbachia* infection status, and major chromosomal inversions. For that purpose, we used a GWAS (Ansari et al. 2017; Fellay and Pedergnana 2020), considering the cryptic mtDNA lineage (vs. the others) discovered in the phylogenetic analyses and how it is associated with each SNP in the nuclear genome. This design aimed to assessing the extent of functional associations between mtDNA lineages and the nuclear genome, highlighting potential mitonuclear coevolution history, considering covariates, such as population structure, *Wolbachia* infection status, and sex.

Following standard practices in GWAS (Uffelmann et al. 2021), we first ensured that the samples included in the Ag1000G were not too closely related. Therefore, we estimated the within-population kinship coefficients using KING 2.2.4 (Manichaikul et al. 2010). The KING-robust approach relies on relationships inference using high density SNP data to model genetic distance between pairs of individuals as a function of their allele frequencies and kinship coefficient (Manichaikul et al. 2010). This contrast with other methods such as the one implemented in PLINK (Purcell et al. 2007) which estimates relatedness using estimator of pairwise identity-by-descent. However, this method is very sensitive to population demography in contrast to the KING-robust approach.

Following The Anopheles gambiae 1000 Genomes Consortium (2017, 2020), we only used the free-recombining biallelic SNPs (*n* = 1,139,052) from section 15 to 41 Mb of chromosome 3L to estimate pairwise kinship coefficients. This genomic portion avoids nonrecombining centromeric regions and major polymorphic chromosomal inversions on chromosome 2, and the sex chromosome. No downsampling, nor LD pruning, nor any other preprocessing was undertaken on the data, following KING's authors recommendation (Manichaikul et al. 2010). We iteratively removed individuals with the largest number of relationships above 2nd degree relative as estimated by KING. At any step, when two individuals were found to have the same number of relationships, we removed the first individual according to its identifier's alpha-numeric order.

In order to include population structure as a covariate in the GWAS analysis, we conducted a PCA following the same procedure as described in The Anopheles gambiae 1000 Genomes Consortium (2017, 2020). We selected randomly 100,000 biallelic SNPs from the free-recombining part of the genome of *An. gambiae* and *An. coluzzii* on chromosome 3L (from position 15 to 41 Mb). To remain consistent with The Anopheles gambiae 1000 Genomes Consortium (2017, 2020), we followed the same filtering procedure. We performed a LD-pruning using the function *locate_unlinked* from Python's module *scikit-allel* version 1.2.1 (Miles and Harding 2016) to ensure independence among SNPs. Specifically, we scanned the genome in windows of 500 bp slid by steps 200 bp and excluded SNPs with an *r*^2^ ≥ 0.1. This process was repeated five times to ensure most SNPs in LD were removed. A PCA was then performed as described in The Anopheles gambiae 1000 Genomes Consortium (2017, 2020), using *scikit-allel* version 1.2.1 (Miles and Harding 2016). Results were plotted highlighting specimens according to their locality of origin. PC scores were stored and used as covariates in the GWAS analysis.

Prior to the actual GWAS analysis, we assessed the extent of association between the two identified major mtDNA phylogenetic lineages, the specimens' sex, *Wolbachia* infection status, and population structure as estimated by the top six PC axes from the PCA. We also considered the inversion karyotypes as reported by The Anopheles gambiae 1000 Genomes Consortium (2020) and Love et al. (2019). Given the diverse nature of covariables, we calculated a proxy of Pearson's correlation coefficient between variables capable of handling numerical and categorical variable types using the *x2y* metric (Ramakrishnan 2021; Lares 2023). The *x2y* metric performs a linear regression on continuous response variables and a classification procedure on categorical response variables. Then, it uses the calculated model to predict the data based on the independent variable and, finally, estimates a percentage of error in the predictions. As this method does not provide any significance test with a *P*-value, the 95% confidence interval was calculated using 1,000 bootstrap resampling (Ramakrishnan 2021; Lares 2023).

Finally, we conducted the formal GWAS-like analysis to evaluate the associations between the two groups of mtDNA phylogenetic lineages and the nuclear SNP genotype variation, considering the following covariates: the population structure using PC scores, the *Wolbachia* infection status, and sex. We performed the GWAS using the program *SNPTEST* v2.5.4-beta3 (Marchini et al. 2007). The main mtDNA lineage were used as “phenotype values” defined as the phylogenetic mtDNA clusters from the hierarchical clustering of the NMDS analysis. After normalization, we used the PC scores of the PCA obtained from S*cikit-allele* as continuous covariates, *Wolbachia* infection status, and sex as binary covariates. Only unrelated samples (*n* = 1,053) and SNPs with a MAF ≥ 0.01 (*n* = 7,858,575, supplementary table S10, Supplementary Material online) were considered in this analysis. The threshold to assess the significance of the GWAS was defined following a Bonferroni-corrected *P*-value accounting for the number of independent genomic blocks in the genome (0.05/1,139,052 = 4.39 × 10^−8^). The number of independent genomic blocks in our dataset was approximated by the number of independent SNPs as determined with Plink v 1.90 (supplementary table S10, Supplementary Material online). The results of the GWAS were plotted as Manhattan and QQ-plots in R.

We produced a list of genes IDs that contained one or more significant SNPs from the GWAS within the CDS or within 1 kb upstream or downstream from the CDS according to the general feature format file VectorBase-57_AgambiaePEST.gff from *VectorBase* release 57, 2022-APR-21 (Giraldo-Calderón et al. 2015). The list of genes was then used to extract information associated with such genes from *VectorBase*.

## Supplementary Material

evae172_Supplementary_Data

## Data Availability

Short-read data used in this study to assemble mitogenomes come from two consortium projects: the MalariaGEN *Anopheles gambiae* 1000 genomes projects phase-2 AR1 data release (https://www.malariagen.net/data_package/ag1000g-phase-2-ar1/); see also The Anopheles gambiae 1000 Genomes Consortium (2020); and the Anopheles 16 genomes project (Fontaine et al. 2015; Neafsey et al. 2015) (National Center for Biotechnology Information, NIH, BioProject IDs: PRJNA67511 and PRJNA254046). Individual NCBI accession IDs are provided in supplementary tables S1 and S2, Supplementary Material online. Mitochondrial genome sequences, mitogenome alignments, related documentations, and scripts are available in DataSuds repository (IRD, France) at https://doi.org/10.23708/WIAJDN. The *AutoMitoG* pipeline, codes, and scripts used in this study are available via GitHub (https://github.com/jorgeamaya/automatic_genome_assembly and https://github.com/jorgeamaya/malaria_mitogenome).
